# A proposed mechanism for intracranial venous lake thrombosis in patients with intracranial signs of hypotension after dural instrumentation

**DOI:** 10.3389/fneur.2025.1588022

**Published:** 2025-07-02

**Authors:** Anna Falk Delgado

**Affiliations:** ^1^Department of Clinical Neuroscience, Karolinska Institutet, Stockholm, Sweden; ^2^Department of Neuroradiology, Karolinska University Hospital, Stockholm, Sweden

**Keywords:** venous thrombosis, intracranial venous lake thrombosis (IVLT), sinus, epidural anesthesia, intracranial hypotension, headache, venous lakes, cerebral venous thrombosis (CVT)

## Abstract

**Introduction:**

Dural sinus venous thrombosis in postpartum women is a well known complication, but intracranial venous lake thrombosis (IVLT) has not been previously described, nor its association with intracranial hypotension following epidural anesthesia (EDA). This study aims to describe and characterize a cohort of patients with IVLT with regards to imaging findings and symptoms.

**Materials and methods:**

This retrospective study included patients from the picture and archiving communication system based on search strategies from referral text including: “headache + EDA”, “complicated EDA”, “post dural puncture headache”, “childbirth + headache”, “delivery + headache” between November 2005 and June 2024. Retrieved examinations were screened for IVLT, intracranial venous thrombosis, and radiological signs of intracranial hypotension. Patient data were extracted and presented descriptively.

**Results:**

Out of 201 patients with 300 investigations, 12 patients (12/201, 6%) had imaging findings suggestive of IVLT. Out of these, 83% (10/12) were in the postpartum period and had received an EDA during delivery, with three (3/12, 25%) stating in the referral that the EDA had been complicated to obtain, with multiple attempts. The mean (SD) Bern score was 6.25 (1.22), indicating a high risk for dural leak. Imaging findings of IVLT included high attenuating thrombotic structures in the parasagittal venous lakes on the inside of the skull convexity on CT with lack of contrast media filling at venous CT-angiography.

**Conclusion:**

We propose a mechanism for intracranial venous lake thrombosis (IVLT) in postpartum females with headaches after complicated EDA with signs of intracranial hypotension.

## Introduction

The intracranial venous system is responsible for draining the brain and intracranial extracerebral structures such as the meninges. Further, it communicates with the extracranial venous system through venous channels in the skull (1), regulates intracranial volume and pressure (2), and contributes to cerebrospinal fluid circulation (3). However, intracranial venous lakes have been given little attention. They receive venous blood from meningeal and diploic veins draining the skull and meninges into the superior sagittal sinus in a one-way fashion (4–7). Intracranial venous thrombosis is a well-known but rare disease (8); however thrombosis localized to the intracranial parasagittal venous lakes—and its potential association with dural tear in the postpartum setting—has not been previously described.

During uncomplicated delivery in uniparous women of first-time deliveries, 7% will have an EDA (9), increasing to 42% with vacuum or forceps extraction. Owing to the potential risk of a dural puncture during complicated EDA, and the risk of cerebrospinal fluid leakage from a dural puncture, changes in cerebrospinal fluid pressure and volume will alter the morphology and volume of the venous system (10), causing traction in the vessel structures and slower venous flow (11, 12). Typically, a cerebrospinal fluid leakage will present clinically as a headache relieved by recumbent position, and exaggerated by standing (13) with brain MRI findings showing enlarged venous structures, dural thickening or subdural fluid collections or hemorrhage. Post dural puncture headache is a known complication after LP (14) and EDA (15). Additionally, postpartum females experience a transitory pro-coagulatory state after delivery (16, 17). Due to complaints of atypical headaches post labor, investigation with brain imaging methods such as CT and MRI can be performed (18).

Cerebrospinal fluid pressure and venous flow are closely linked systems (2). In intracranial hypotension, intracranial venous structures will present with compensatory dilation (19). Since venous structures have wide dissemination in dural structures (20, 21), patients with intracranial hypotension will show a characteristic general dural dilatation including the cerebellar tentorium, the falx cerebri and under the convexity, sometimes even leading to subdural hemorrhage (22). On imaging, acute thrombosis is known to present with high density at CT in the first days after symptom onset (23–25) but can be missed close to the skull bone due to streak artifacts near bony structures.

This work describes a proposed mechanism for a new entity—intracranial venous lake thrombosis (IVLT)—here presented in a retrospective cohort of postpartum women after delivery with EDA and intracranial hypotension, as well as in other patients with intracranial hypotension and pro-coagulatory risk factors.

## Materials and methods

Patients from the Picture and Archiving Communication System (PACS) were retrospectively included in this cohort study, after study approval from the Swedish Ethical Review Authority in March 17th 2024, number 2024-00249-02. Informed consent was waived due to the retrospective nature of the study. Patients were eligible for study inclusion if they were retrieved in the search strategy. The following search terms were used to search for text in previous radiological referrals: “headache + EDA”, “complicated EDA”, “post dural puncture headache”, “childbirth + headache”, “delivery + headache” between November 2005 and June 2024. The search term “post dural puncture headache” was specifically included to retrieve cases of both sexes and varied clinical history not necessarily including a history of childbirth. Both CT and MRI investigations were included. Further, patients with IVLT known from clinical work, were collected from the PACS. All retrieved examinations from the search strategies were screened for signs of IVLT and other intracranial venous thrombosis by one specialist in Neuroradiology with 10 years experience of working in a neuroradiology unit. For examinations where IVLT was suspected, data was extracted according to a prespecified study protocol, including Bern score (10), imaging findings of subdural hematoma, widened venous structures, and cortical-, sinus-, or venous- lake thrombosis. Further, information on patient age (years), sex (female/male/transgender), days between delivery/lumbar puncture and radiological investigation, headache (Yes/No), EDA/LP (Yes/No), postpartum (Yes/No), and if intracranial venous thrombosis had been previously described in the report text (Yes/No) were collected. For patients with suspected IVLT, electronic health records and radiological follow-ups were screened for assessment of previous treatment, resolution of clinical symptoms and imaging findings.

## Results

**Figure 1:**
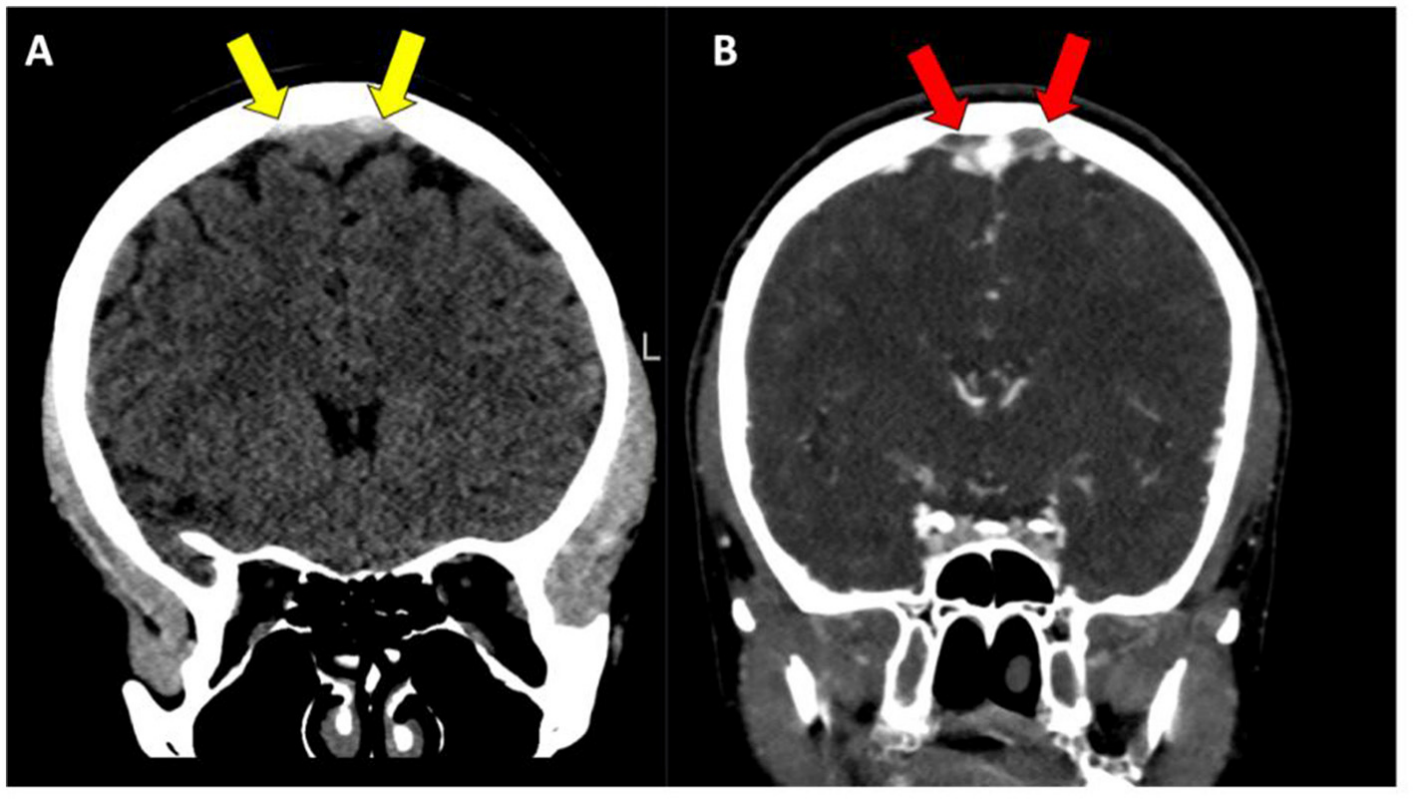
**(A, B)** Patient with parasagittal intracranial venous lake thrombosis (IVLT), with high attenuating thrombosis located on both sides of the superior sagittal sinus on non-contrast enhanced CT images (yellow arrows), with a corresponding lack of filling in the same venous lake structures on CT-venography (red arrows).

**Figure 2:**
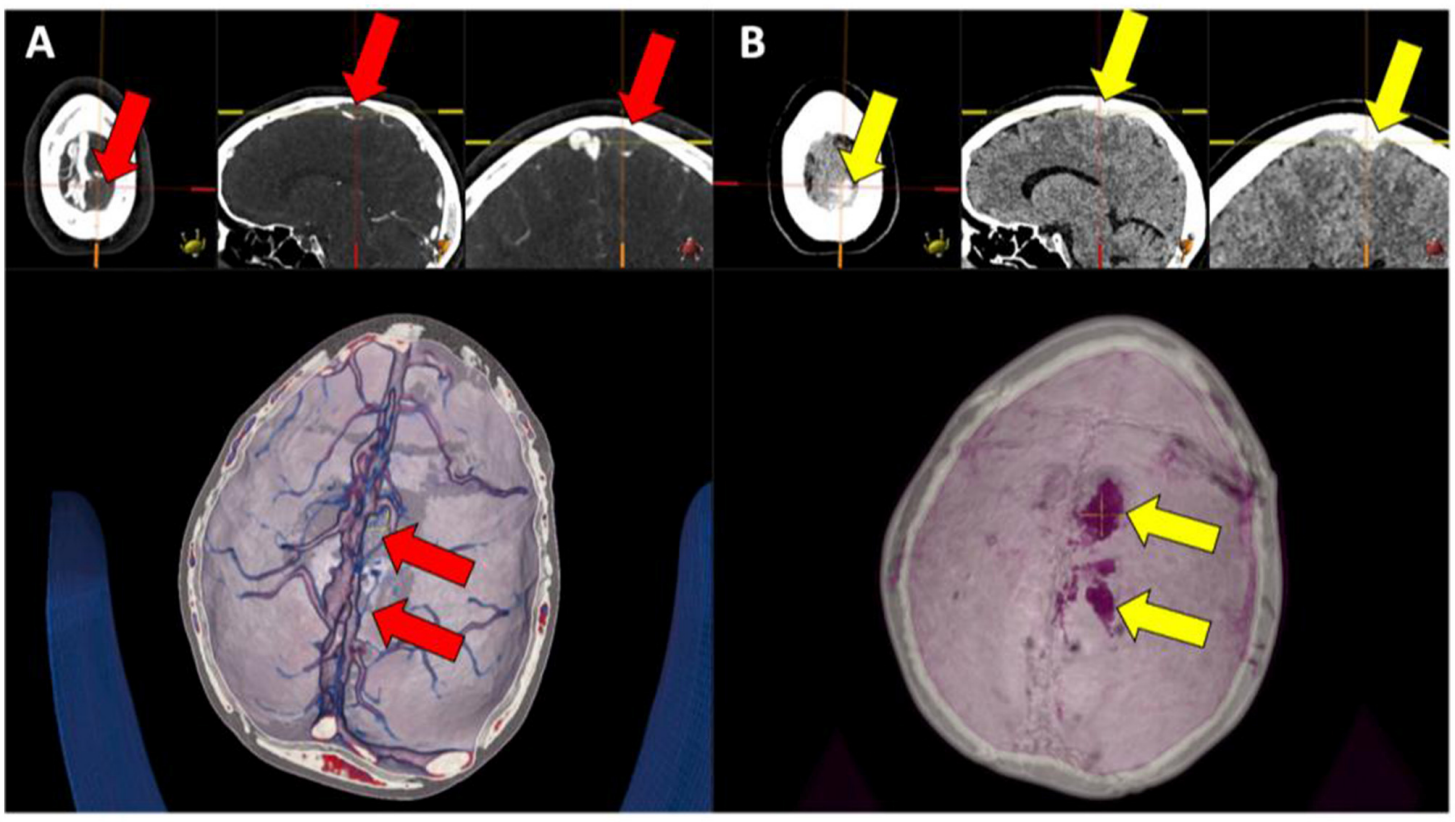
**(A, B)** Left parasagittal high attenuating structure (right image set, yellow arrows) in the venous lakes, corresponding to a lack of contrast media filling of the same engorged venous lake structures (left image set, red arrows).

In total, the searches yielded 201 patients with 300 investigations that were screened for imaging findings of IVLT. Twelve patients (12/201, 6%) had imaging findings suggestive of IVLT (Supplementary materials). Additional search terms did not yield any additional cases. Out of these, a majority were in the postpartum period 10/12, 83%, and had received an EDA during delivery, with three (3/12, 25%) stating in the referral that the EDA had been complicated to obtain, with multiple attempts. The mean (SD) Bern score was 6.25 (1.22), indicating a high risk for dural leak. Detailed results of Bern score can be found in Table 1. Basic demographics for patients with IVLT are summarized in Table 2. The mean age was 31 (SD 4), and 92% (11/12) were female. One person was transgender (female-to-male) under testosterone treatment with high-dose cortisone due to suspected neuroinflammatory disease (1/12, 8%). One patient was pregnant, undergoing lumbar puncture due to suspected intracranial hypotension. Initial imaging investigations were performed at a mean of 3 days (SD 2) after delivery/LP. Figures 1, 2 shows typical appearances on imaging of IVLT.

**Table 1:** BERN score.

**ID**	**1**	**2**	**3**	**4**	**5**	**6**	**7**	**8**	**9**	**10**	**11**	**12**
Engorgement of venous sinus	2	2	2	2	2	2	2	2	2	2	2	2
Pachymeningeal enhancement	2	NA	NA	NA	NA	NA	NA	2	NA	NA	NA	NA
Suprasellar cistern (≤ 4 mm)	2	2	2	2	2	2	2	2	2	2	2	2
Subdural fluid collection	1	0	0	1	1	0	1	0	1	1	0	0
Prepontine cistern (≤ 5 mm)	1	0	0	1	1	1	0	1	1	1	1	1
Mamillopontine distance (≤ 6.5 mm)	1	1	1	1	1	0	0	0	0	1	1	1
Sum BERN score:	9	5	5	7	7	5	5	7	6	7	6	6

**Table 2:** Patient characteristics.

**ID**	**Sex (Female F/Male M)**	**Age**	**Postpartum**	**Days between delivery/LP and imaging**	**EDA or LP**	**Venous lake thrombosis**	**Suspicion of thrombosis on original report**	**Bern score**
1	F	32	Yes	5	EDA	Yes	No	9
2	F	35	Yes	2	EDA	Yes	Yes (cortical veins)	5
3	F	28	Yes	7	EDA	Yes	No	5
4	F	34	Yes	3	EDA	Yes	Yes (cortical veins)	7
5	F	31	Yes	3	EDA	Yes	Yes (cortical veins)	7
6	F	25	Yes	3	EDA	Yes	No	5
7	F	29	No (pregnant)	NA (pregnant)	NA	Yes	No	5
8	F	26	Yes	1	LP	Yes	No	7
9	F	26	Yes	4	EDA	Yes	No	6
10	F	29	Yes	4	EDA	Yes	No	7
11	F	37	Yes	3	EDA	Yes	No	6
12	F-to-M transgender	38	No	3	LP	Yes	Yes (cortical veins)	6

In the IVLT group, only 4/12, 33% were diagnosed with possible intracranial venous thrombosis at the time of investigation, with none mentioning the possibility of venous lake thrombosis in the report. However, cortical vein thrombosis was the most common initial diagnosis at the time of investigation (4/12, 33%). Other imaging findings were thin fluid collections located near the cerebellar tentorium. A total of 100% of included patients reported headaches. On re-evaluation of the images, one patient had a small thrombosis at the inflow from the inferior anastomotic vein into the right transversal sinus, and one had a small thrombosis involving a focal part of a lateral compartment of the superior sagittal sinus. No patient had massive dural sinus venous thrombosis.

Six patients (6/12, 50%) received treatment for suspected dural leak with epidural blood patch and two patients (2/12, 8%) received treatment for venous thrombosis (Heparin or Fragmin). Patients had undergone clinical evaluation for up to a mean duration of 52 months (SD 64). One patient had died 4 years after diagnosis due to an acute hematological disorder. Three of the 12 patients (25%) had identifiable risk factors for venous thromboembolism: protein S deficiency, testosterone/cortisone therapy, and rheumatoid arthritis. Seven (7/12, 58%) patients were followed-up with imaging (CT or MRI) after a mean (SD) time of 8 (8) months with resolution of imaging findings (IVLT and intracranial hypotension) and symptoms.

**Figure 3:**
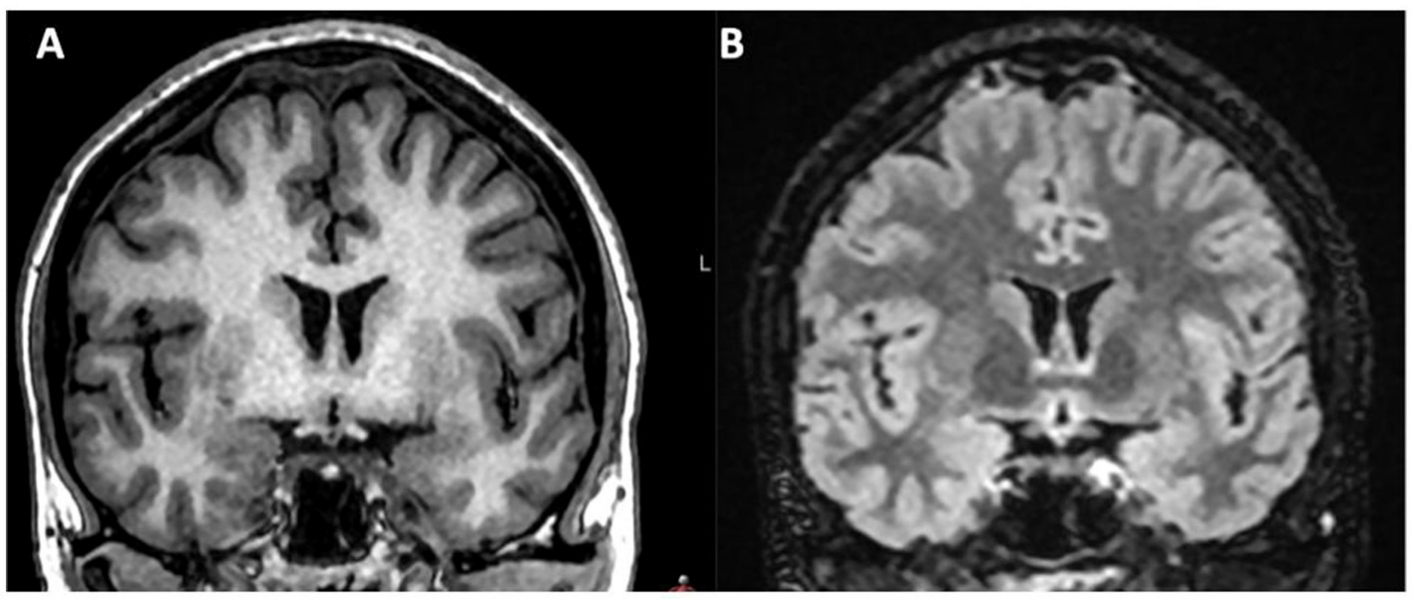
**(A, B)** Patient not presenting with IVLT after delivery with EDA, but signs of intracranial hypotension, with dilatation of parasagittal venous lakes. Coronal T1 MRI image and coronal T2 FLAIR image.

**Figure 4:**
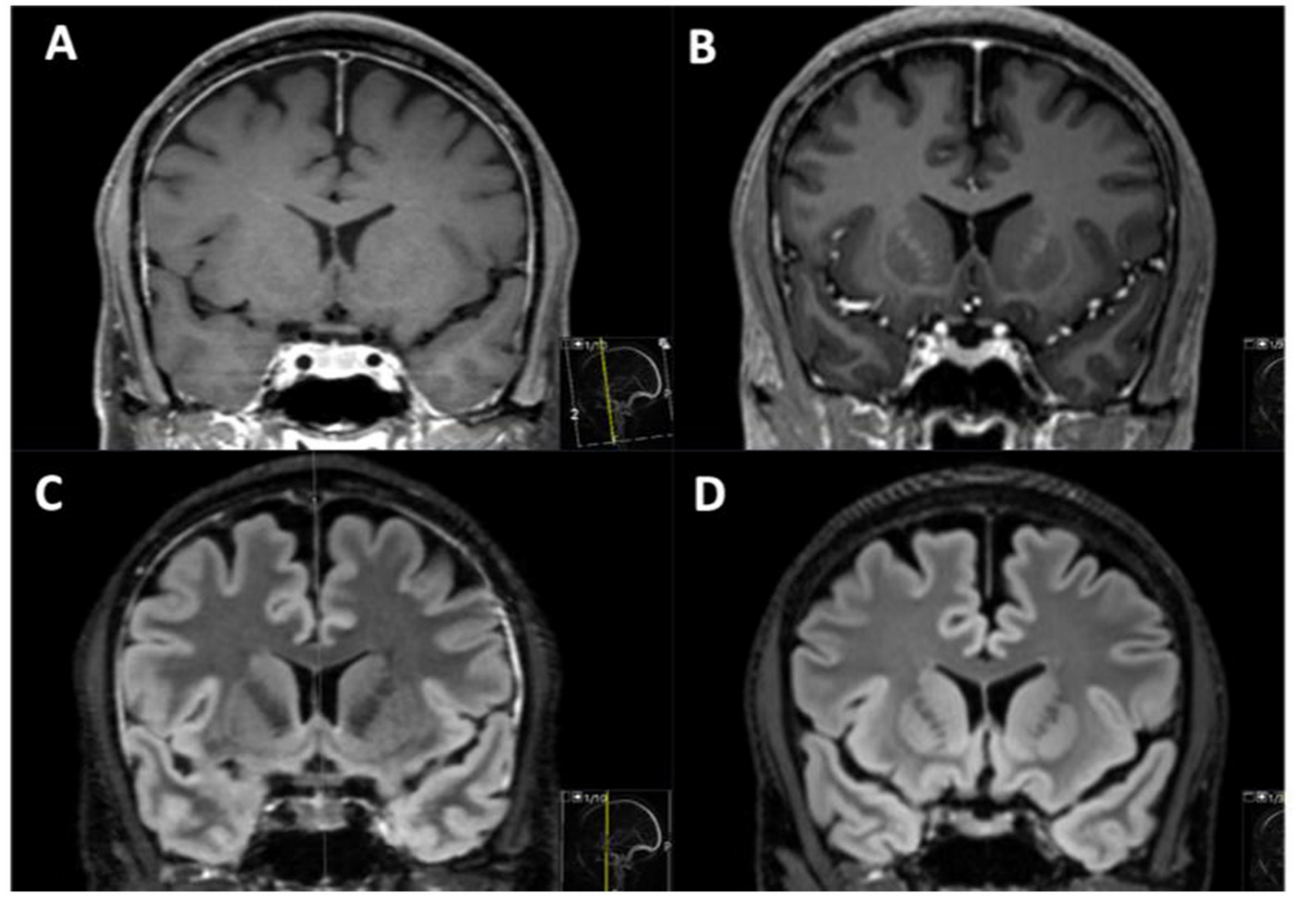
MRI in one patient without venous lake thrombosis investigated before **(B, D)** and after **(A, C)** lumbar puncture to investigate headache. After lumbar puncture, thickened dural enhancement can be noted on T1 Gd images **(A)**, and an increased signal on T2 FLAIR **(C)** in the widened dura can be observed, with an increase in pituitary eight **(A, C)**.

In patients without IVLT, retrospective analysis identified only one patient (male) with concurrent cortical vein thrombosis one day after lumbar puncture for optic neuritis, and 13 patients with thickened dura or thin subdural hematoma after LP, EDA or spinal anesthesia compatible with intracranial hypotension. Hence, no patient had isolated dural sinus venous thrombosis. One patient had engorged rounded veins without a history of iatrogenic dural puncture, but findings compatible with spontaneous intracranial hypotension. Figures 3, 4 shows imaging examples of patients with intracranial hypotension without venous thrombosis.

## Discussion

This retrospective cohort study describes a new entity of intracranial venous thrombosis located specifically in the intracranial venous lakes, IVLT, that has not been previously described. Postpartum women with a history of EDA and headache after delivery, as well as other persons with intracranial hypotension and pro-coagulatory risk factors can present with IVLT. Specific imaging findings include high attenuating thrombotic material in the venous lakes close to the superior sagittal sinus, and simultaneous loss of contrast enhancement on venous angiography.

This study highlights another possible adverse effect of intracranial hypotension after EDA with unintentional dural puncture in postpartum women. Although it can also affect patients with dural leak from other causes, such as LP. Study findings can guide future radiological imaging, image reading, and reporting on these structures close to the calvarium, often missed due to the prevalence of high attenuating streak artifacts close to the bone. Adding a CT or MRI venography to the non-enhanced examination in our cohort added valuable diagnostic information in the retrospective evaluation by strengthening the suspicion of thrombosis. IVLT differs from previously recognized intracranial thrombotic states in terms of probable pathophysiological mechanism and thrombotic location.

Although the pathophysiological mechanism remains to be further elucidated, it is probably related to the pro-coagulatory state in this specific patient group, with the co-occurring dilatation of venous lakes due to intracranial hypotension. It is currently unknown how the venous dilatation leads to the formation of a thrombus, but as proposed by other studies, flow mechanism and stagnant blood in locations with venous junctions may play a contributing role (12). Further, one hypothesis is that morphological rearrangement caused by intracranial hypotension might play a greater role at the parasagittal convexity, with venous routes possibly hindered or focally injured when traction between tissues occur. Proximity to parietal foramina could also matter. The proposed pathophysiological mechanism is further supported by the fact that IVLT can occur in any patient with intracranial vein engorgement and pro-coagulatory risk factors such as in the two patients in our cohort who had not recently undergone a delivery. Notably, in our cohort, all patients with IVLT had a history of epidural anesthesia or lumbar puncture, suggesting a strong association between IVLT and prior dural instrumentation.

Recent studies have visualized the parasagittal venous lakes draining the dura and proposed an association with arterio-venous fistulas, highlighting other diseases possibly related to these structures (6). Further, previous studies have reported on the association of dural venous sinus thrombosis and intracranial hypotension (11, 12, 26), but none have reported on thrombosed intracranial venous lakes. Although post-lumbar puncture intracranial hypotension syndrome has been previously described, it does not entail IVLT (27, 28). Further, one study has highlighted a possible association between epidural blood patch and cerebral venous thrombosis (29) indicating the unclear association between hypotension vs. its treatment in relation to intracranial venous thrombosis.

Study limitations are attributed to the non-standardized imaging regimen in retrospective data. However, it was also beneficial, to be able to visualize IVLT on both CT and MRI, and on different scanners, thereby increasing the generalizability of the findings. Although linked to the fact that many patients did not get an initial diagnosis, another limitation was the lack of standardized follow-up. However, in patients investigated longitudinally, imaging findings of IVLT resolved together with the symptoms. The lack of pathological confirmation of findings is related to the benign prognosis but also makes the possibility for the findings to be related to focal hemorrhage a yet unstudied topic.

Although an underdiagnosed and hitherto unrecognized entity, IVLT appears to possibly have a more benign course than other intracranial venothrombotic states such as cortical venous thrombosis and dural sinus venous thrombosis (30), potentially it could be an incidental finding. However, since patients present with atypical headaches necessitating radiological work-up there is a clinical need for diagnosis and patient information in this subclassification of intracranial venous thrombosis. Although potentially treatable with anticoagulant therapy, the lack of recognition at presentation in this retrospective cohort highlights that most patients were dismissed and returned home without specific anticoagulation treatment or standardized imaging follow-up. This highlights the possible role of the body's own thrombolytic regulatory mechanisms and restitution after delivery with normalization of coagulatory status but will need to be confirmed in prospective studies. The best medical treatment for IVLT is currently unclear, but a majority of the included patients received treatment for intracranial hypotension with epidural blood patch. A couple of patients also received anticoagulatory medication due to suspected cortical vein thrombosis (31). A prophylactic treatment regimen with low molecular weight heparin, compression socks, and intermittent pneumatic compression, such as for deep venous thrombosis of the legs has not yet been investigated (32).

All included patients had improved at follow-up, and no one deteriorated from IVLT. Since the search strategy strove to include patients with clinical signs of hypotension, the prevalence of IVLT in other groups has not been evaluated. However, from clinical experience this has not been an evident finding in other patient groups. Study findings indicate a need for risk factors screening to possibly prevent future potential thromboembolism.

Future studies should focus on confirming imaging findings of IVLT, for example by performing multi-phase angiography studies of the venous lakes and longitudinal follow-up of signal changes inside the venous lakes. Prospective studies should focus on the diagnosis, optimal treatment, and follow-up of IVLT compared to controls. Additionally, a multicenter study aggregating patients nationwide would further increase knowledge and deeper characterization of this disease. Future prospective studies should investigate if headaches caused by IVLT and intracranial hypotension differ from headaches caused by intracranial hypotension alone.

## Conclusion

In conclusion, we propose a mechanism for intracranial venous lake thrombosis (IVLT) in postpartum females with headaches after complicated EDA with signs of intracranial hypotension.

## Data Availability

The original contributions presented in the study are included in the article/Supplementary material, further inquiries can be directed to the corresponding author.
