# Supplementary material for: A proposed mechanism for intracranial venous lake thrombosis in patients with intracranial signs of hypotension after dural instrumentation
Source: Front Neurol. 2025 Jul 2;16:1588022. doi: 10.3389/fneur.2025.1588022 (PMC12263696; doi:10.3389/fneur.2025.1588022)
Supplement: Supplementary file 1 [file Data_Sheet_1.pdf]

Supplemental Materials

Images on intracranial venous lake thrombosis (IVLT)

ID 1.

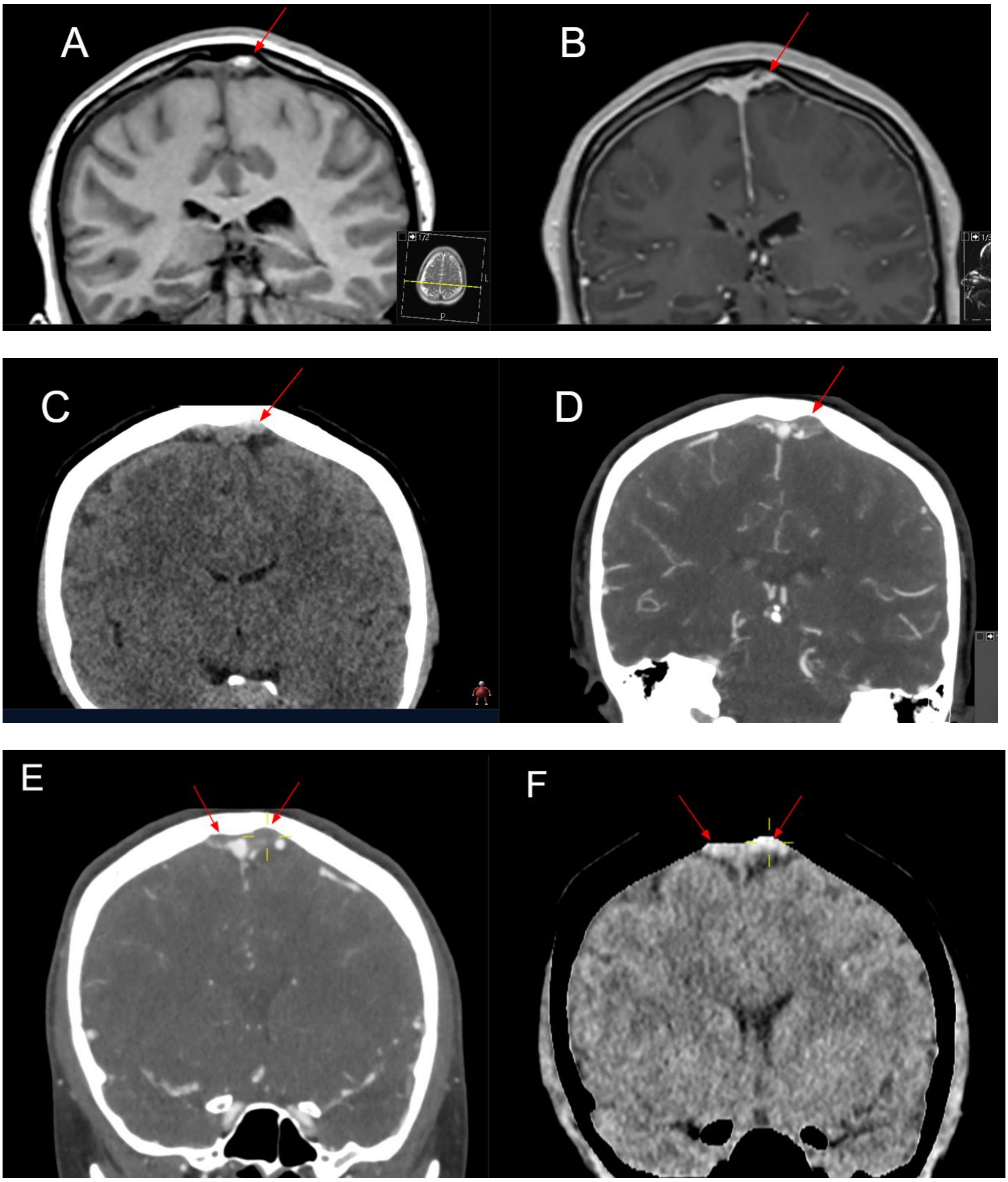

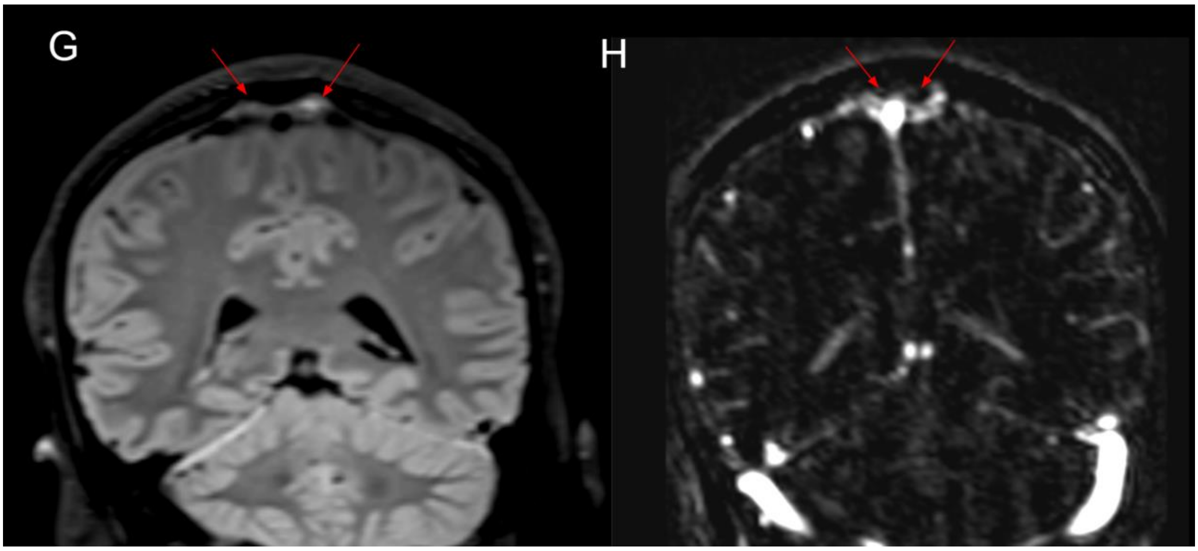

A. MRI T1 with red arrow indicating high signal in thrombus
B. MRI T1 post gadolinium with red arrows indicating loss of contrast filling at the site of the thrombus
C. Non-enhanced CT with red arrow indicating high attenuating thrombus
D. CT venography with red arrow indicating lack of filling at the site of the thrombus
E. CT venography with red arrows indicating lack of filling at the site of the thrombus
F. Non-enhanced CT with red arrow indicating high attenuating thrombus
G. MRI T2 FLAIR with red arrows indicating high signal in thrombus
H. MRI venography with red arrows indicating lack of flow at the site of thrombus

ID 2.

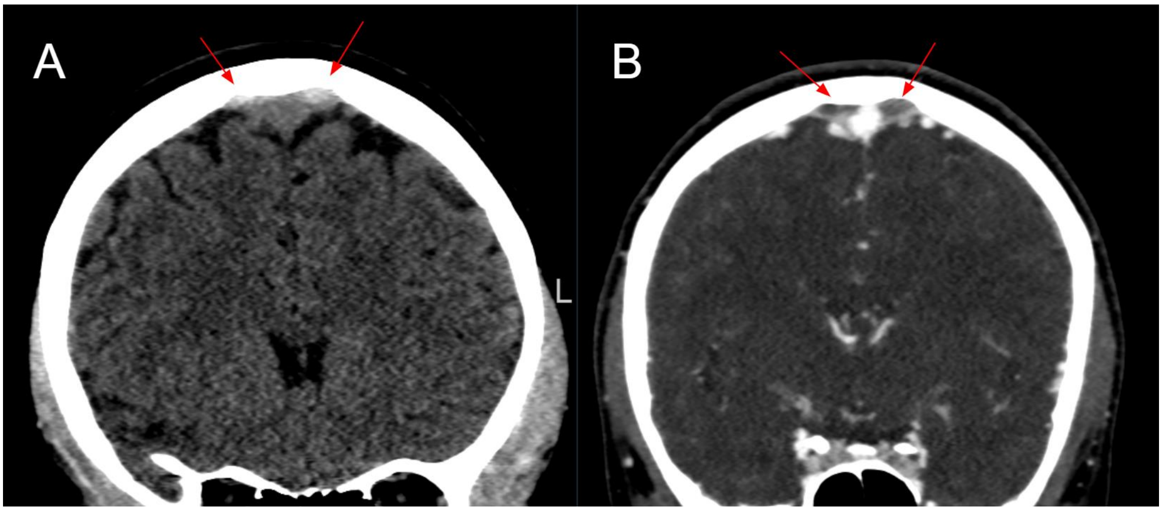

A. Non-enhanced CT with red arrows indicating high attenuation in the thrombus
B. CT venography with red arrows indicating lack of filling at the site of thrombus

ID 3.

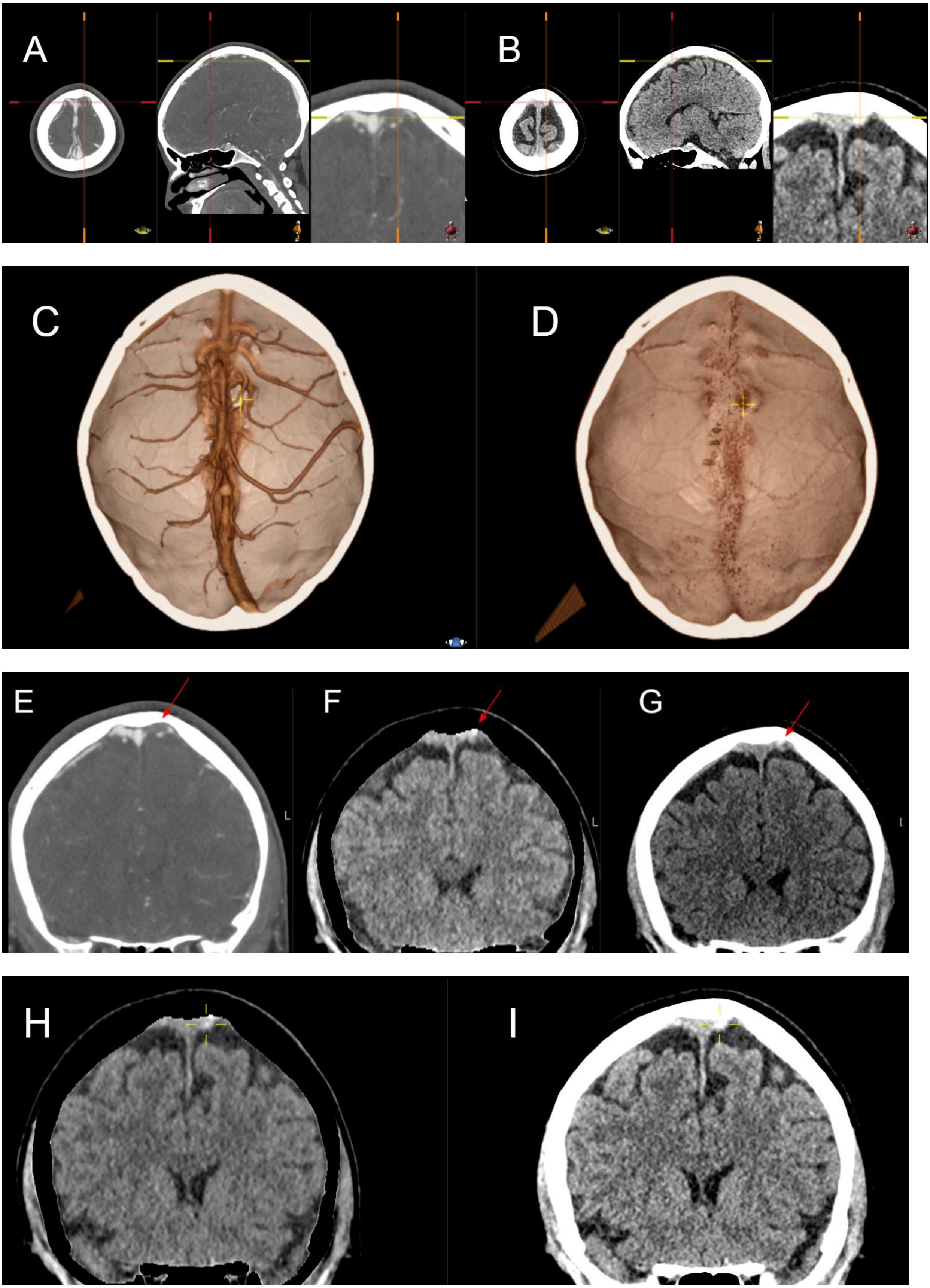

A. CT venography with cross hair indicating lack of filling at the site of the thrombus

B. Non-enhanced CT with cross hair indicating high attenuation at the site of the thrombus
C. Volume rendering of CT venography with cross hair indicating lack of filling at the site of the thrombus
D. Volume rendering of non-enhanced CT with cross hair indicating high attenuation at the site of the thrombus
E. CT venography with red arrow indicating lack of filling at the site of thrombus
F. Non enhanced CT (bone removed) with red arrow indicating high attenuation in the thrombus
G. Non-enhanced CT with red arrow indicating high attenuation in the thrombus
H. Non-enhanced CT (bone removed) with cross hair indicating high attenuation in the thrombus
I. Non-enhanced CT with cross hair indicating high attenuation in the thrombus

ID 4.

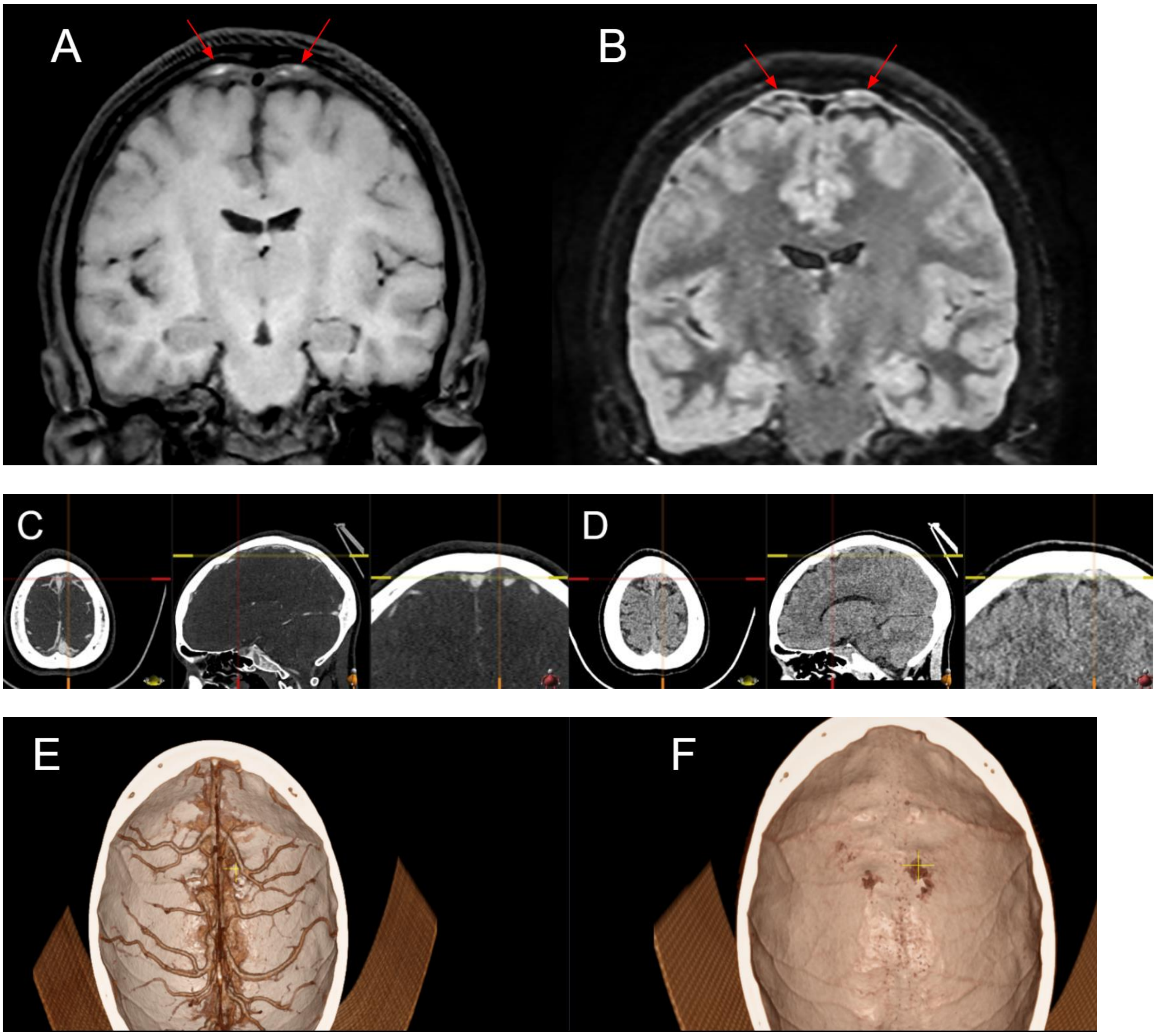

A. MRI T1 with red arrows indicating high signal at the site of thrombus
B. MRI T2FLAIR with red arrows indicating high signal at the site of the thrombus
C. CT venography with cross hairs indicating lack of filling at the site of the thrombus
D. Non-enhanced CT with cross hair indicating high attenuation at the site of thrombus
E. Volume rendering of CT venography with cross hair indicating lack of filling at the site of the thrombus
F. Volume rendering of non-enhanced CT with cross hair indicating high attenuation at the site of the thrombus

ID 5.

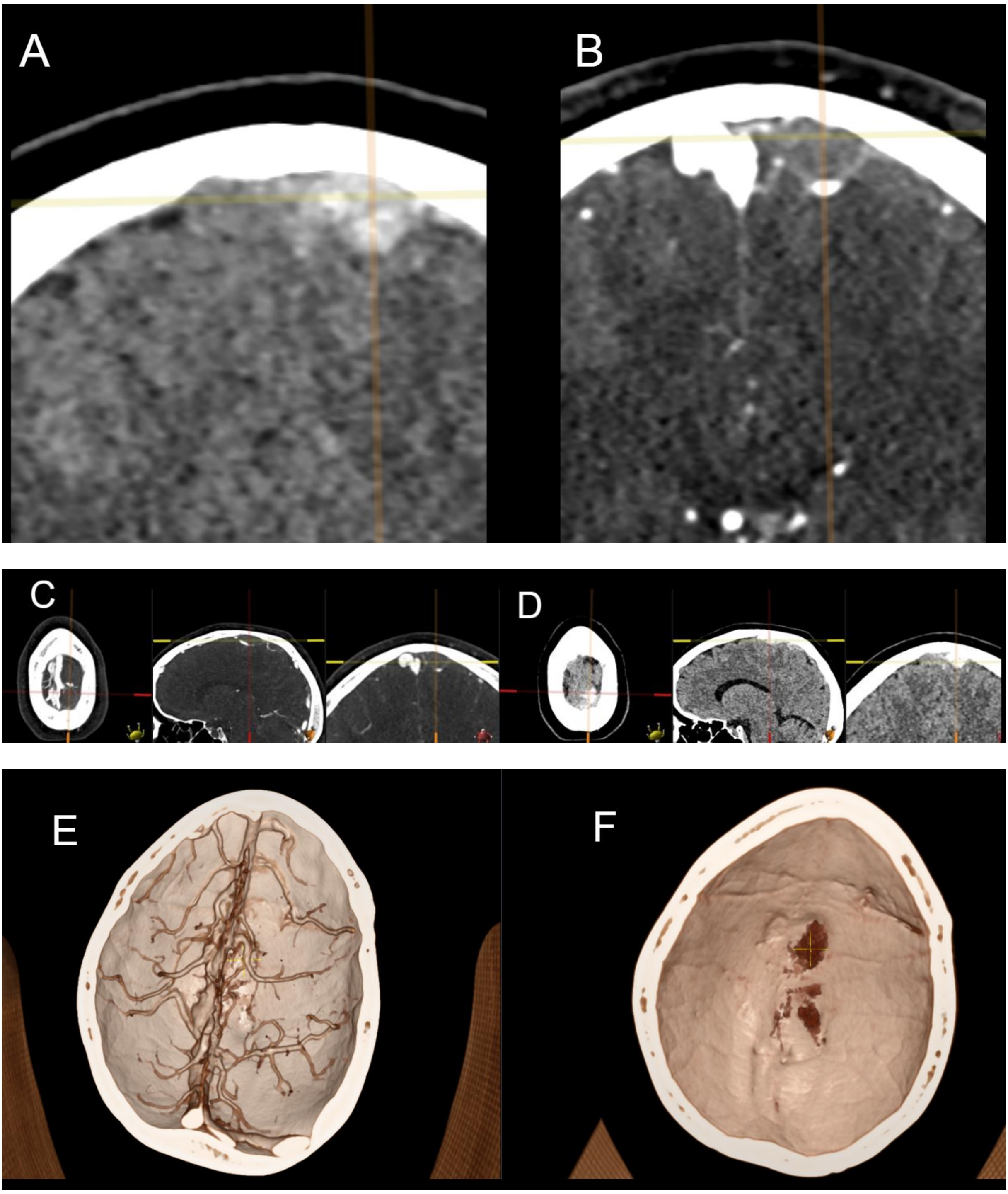

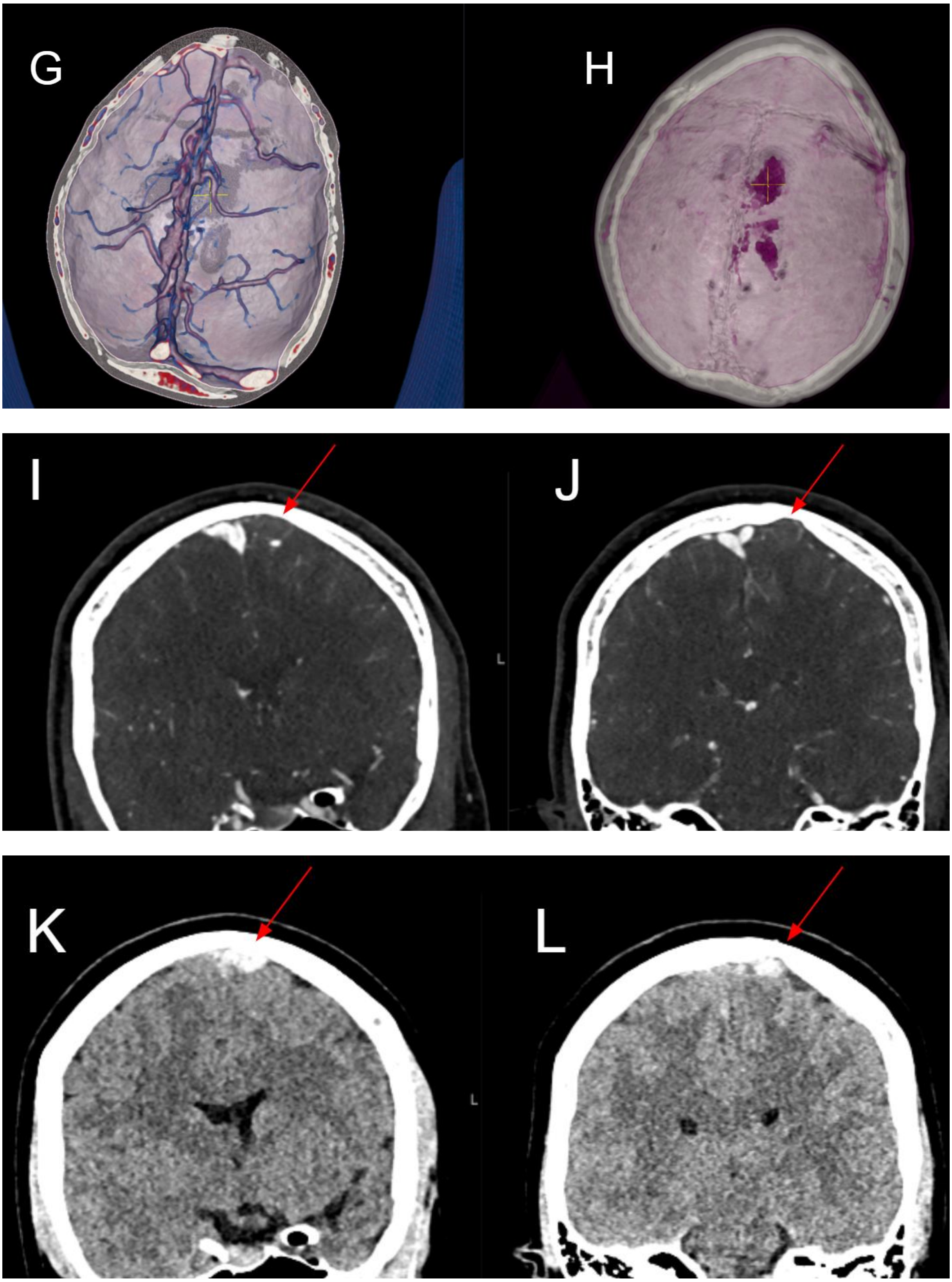
G
H
I
J
L
K
L
L

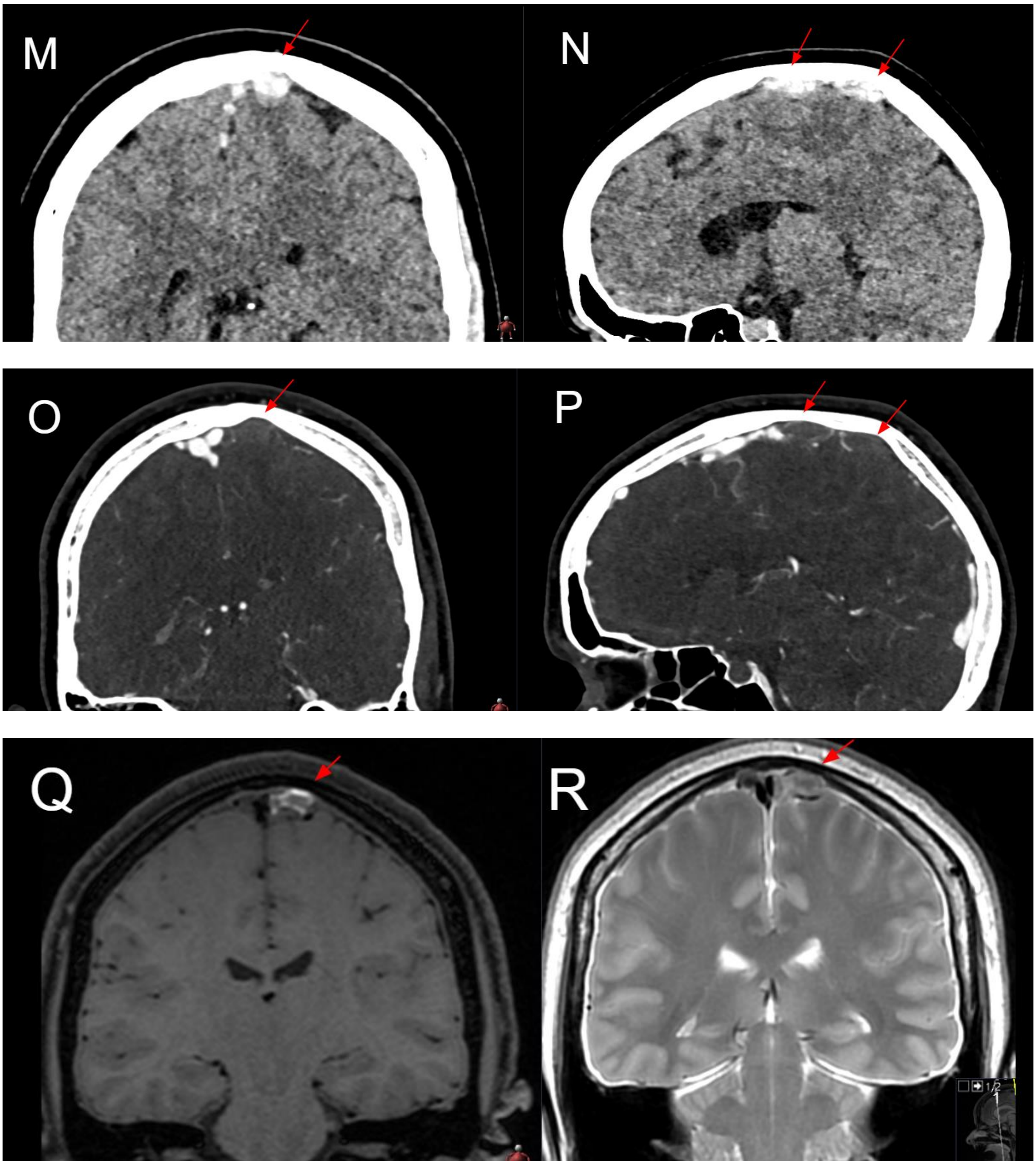
M
N
O
P
Q
R

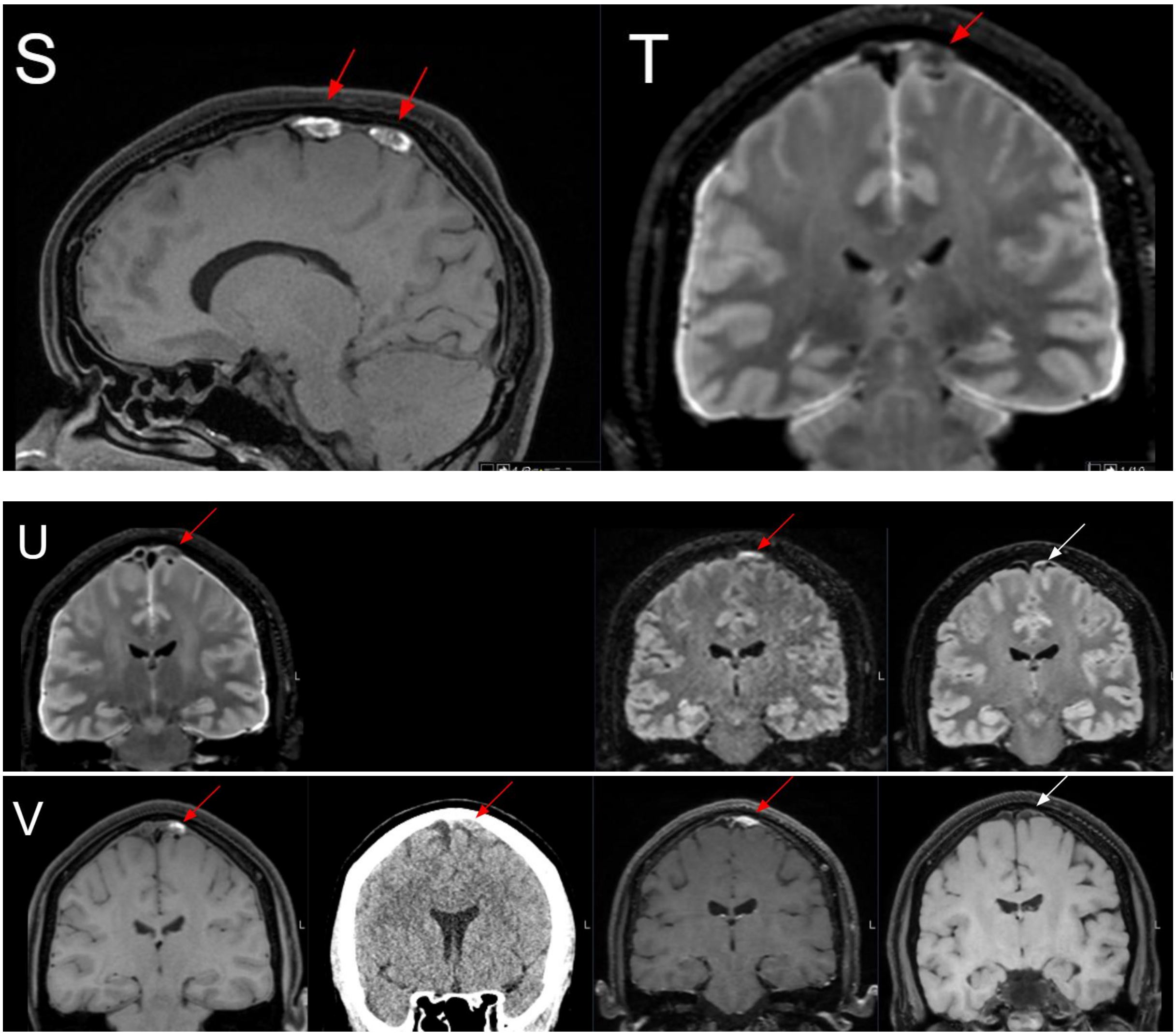

A. Non-enhanced CT with cross hair indicating high attenuation at the site of the thrombus
B. CT venography with cross hair indicating lack of filling at the site of the thrombus
C. CT venography in multiplanar reformation with cross hair indicating lack of filling at the site of the thrombus
D. Non-enhanced CT in multiplanar reformation with cross hair indicating high attenuation at the site of the thrombus
E. Volume rendering of CT venography with cross hair indicating lack of filling at the site of the thrombus
F. Volume rendering of non-enhanced CT with cross hair indicating high attenuation at the site of the thrombus
G. Volume rendering of CT venography with cross hair indicating lack of filling at the site of the thrombus
H. Volume rendering of non-enhanced CT with cross hair indicating high attenuation at the site of the thrombus
I. CT venography with red arrow indicating lack of filling at the site of the thrombus
J. CT venography with red arrow indicating lack of filling at the site of the thrombus
K. Non-enhanced CT with red arrow indicating high attenuating thrombus
L. Non-enhanced CT with red arrow indicating high attenuating thrombus

M. Non-enhanced CT with red arrow indicating high attenuating thrombus
N. Non-enhanced CT with red arrows indicating high attenuating thrombus
O. CT venography with red arrow indicating lack of filling at the site of the thrombus
P. CT venography with red arrows indicating lack of filling at the site of the thrombus
Q. MRI T1 with red arrow indicating high signal in thrombus
R. MRI T2 with red arrow indicating loss of flow vid at the site of the thrombus
S. MRI T1 with red arrows indicating high signal in thrombus
T. MRI T2 FLAIR with red arrow indicating high signal at the site of the thrombus
U. Longitudinal imaging of thrombus involution at MRI T2FLAIR from total filling of high signal thrombus to thin high signal thrombus remnant indicated by the white arrow
V. Longitudinal imaging of thrombus involution at MRI T1 (and one CT) from total filling of high signal thrombus to disappearance of high signal thrombus indicated by the white arrow

ID 6.

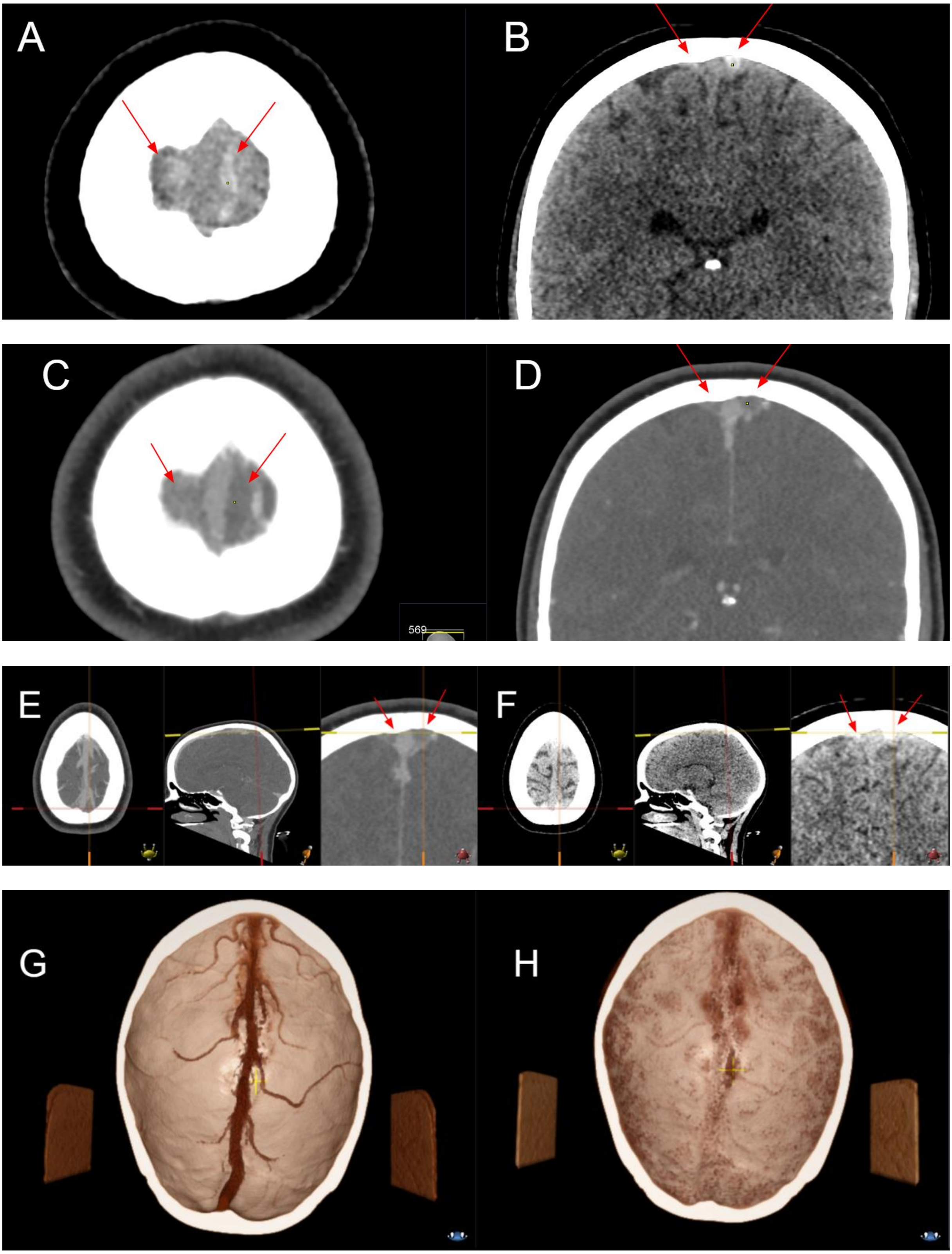

A. Non-enhanced CT with red arrows indicating high attenuating thrombus
B. Non-enhanced CT with red arrows indicating high attenuating thrombus
C. CT venography with red arrows indicating lack of filling at the site of the thrombus
D. CT venography with red arrows indicating lack of filling at the site of the thrombus

E. CT venography in multiplanar reformation with cross hair and red arrows indicating lack of filling at the site of the thrombus
F. Non-enhanced CT in multiplanar reformation with cross hair and red arrows indicating high attenuating thrombus
G. Volume rendering of CT venography with cross hair indicating lack of filling at the site of the thrombus
H. Volume rendering of non-enhanced CT with cross hair indicating high attenuation at the site of the thrombus

ID 7.

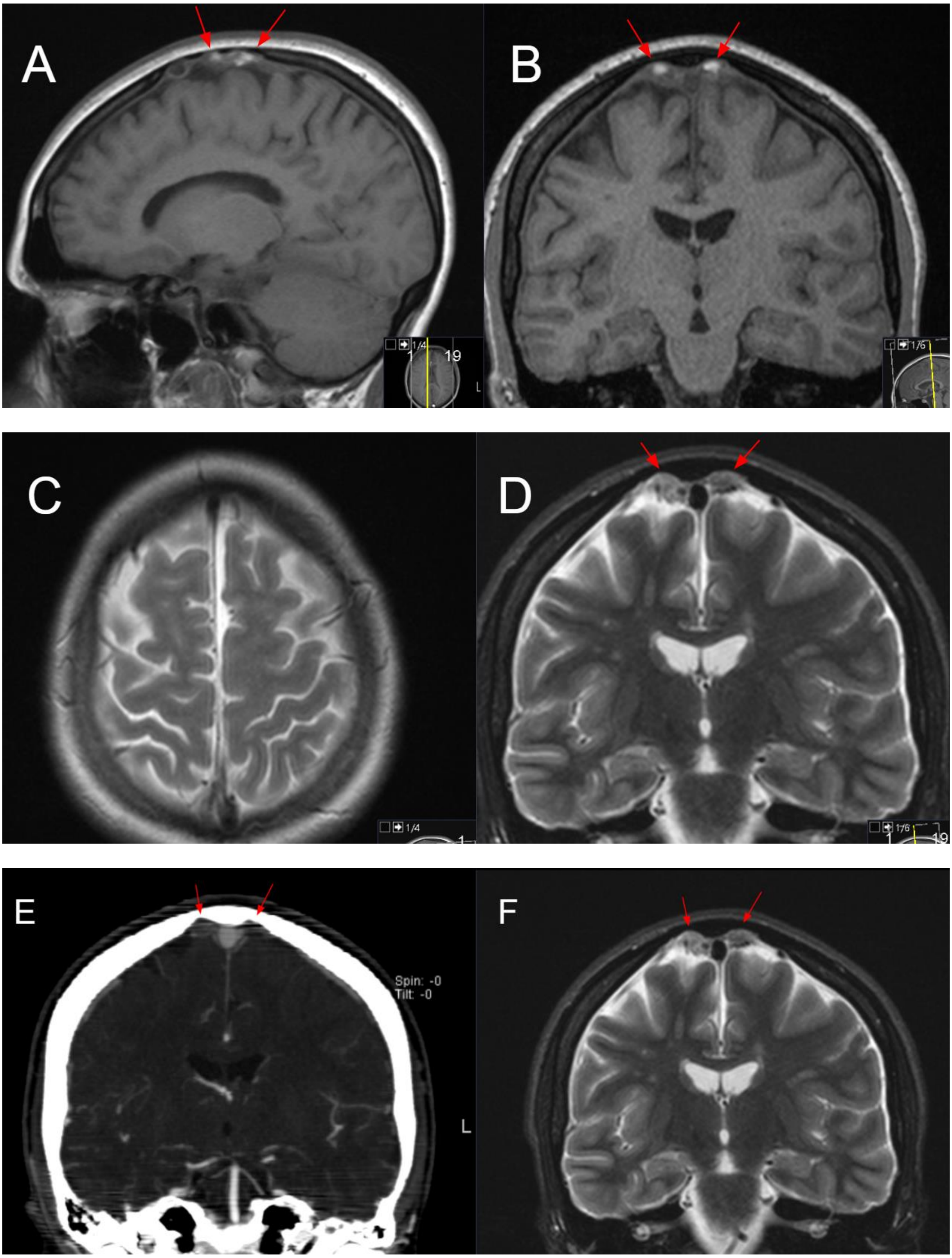

A. MRI T1 with red arrows indicating high signal in thrombus
B. MRI T1 with red arrows indicating high signal in thrombus
C. MRI T2 with signs of subdural fluid collection near falx cerebri
D. MRI T2 with red arrows indicating loss of flow vid at the site of the thrombus

E. CT venography with red arrows indicating lack of filling at the site of the thrombus
F. MRI T2 with red arrows indicating loss of flow vid at the site of the thrombus

ID 8.

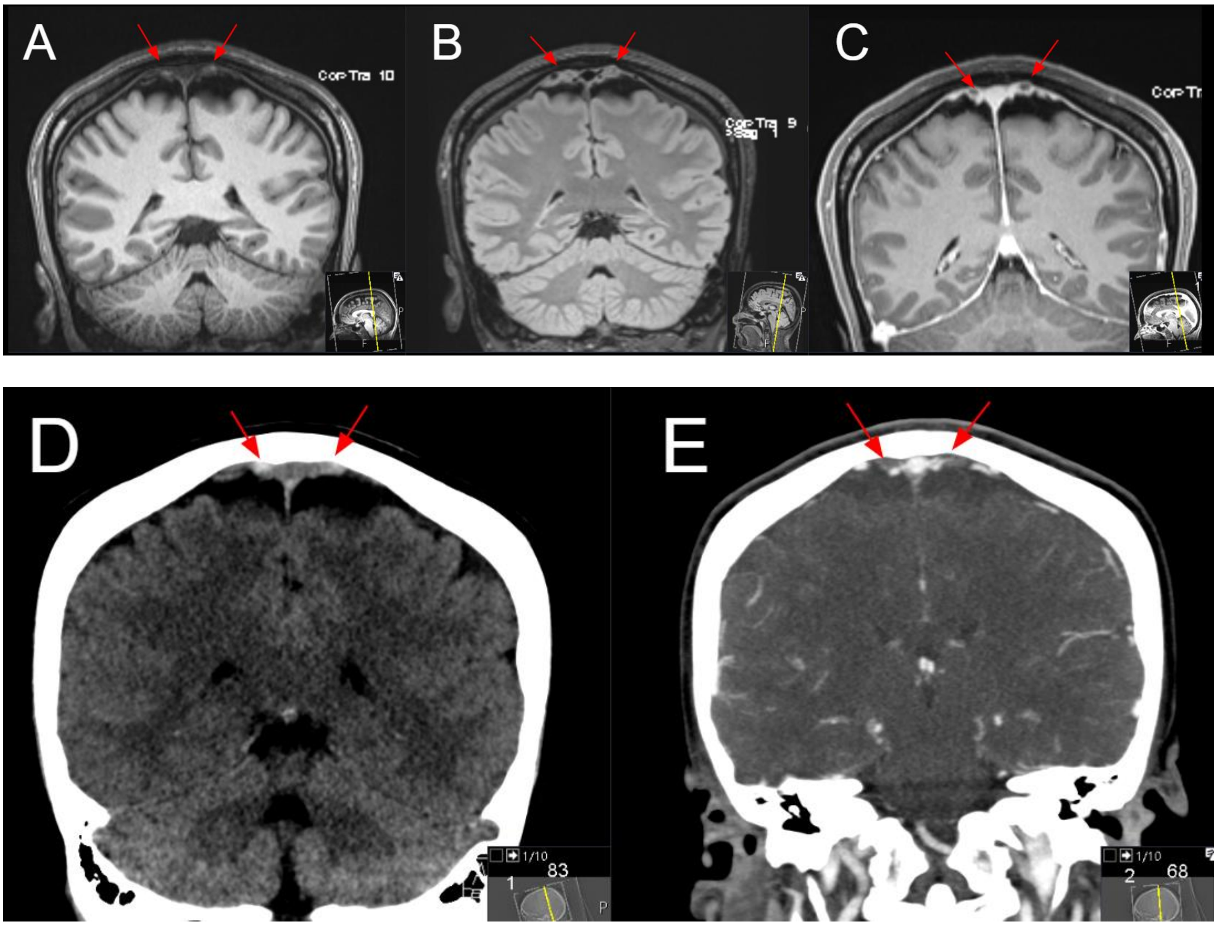

A. MRI T1 with red arrows indicating high signal in thrombus
B. MRI T2 FLAIR with red arrows indicating high signal in thrombus
C. MRI T1 post gadolinium with red arrows indicating loss of contrast filling at the site of the thrombus
D. Non-enhanced CT with red arrows indicating high attenuating thrombus
E. CT venography with red arrows indicating lack of filling at the site of the thrombus

ID 9.

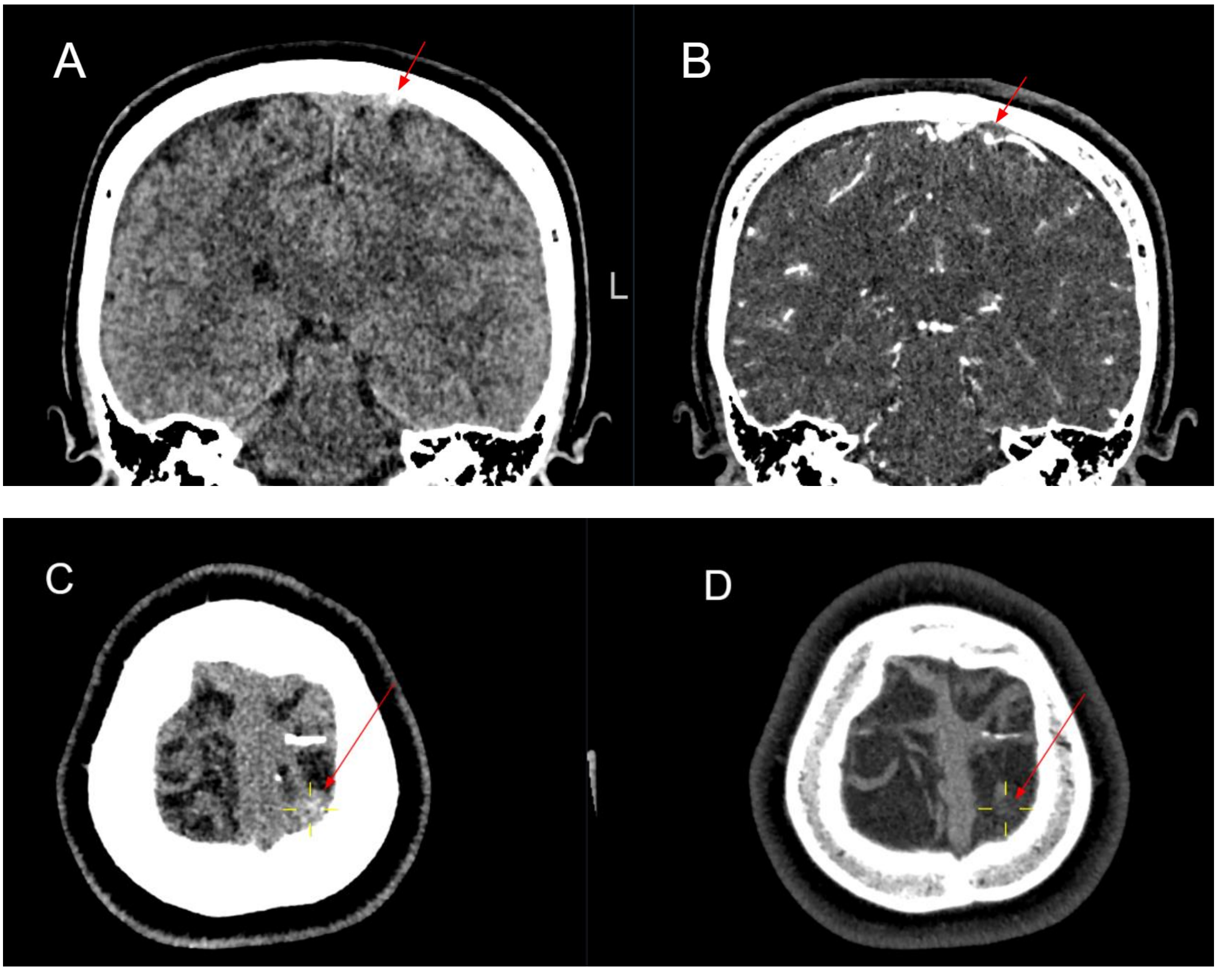

A. Non-enhanced CT with red arrow indicating high attenuating thrombus
B. CT venography with red arrow indicating lack of filling at the site of the thrombus
C. Non-enhanced CT with red arrow indicating high attenuating thrombus
D. CT venography with red arrow indicating lack of filling at the site of the thrombus

ID 10.

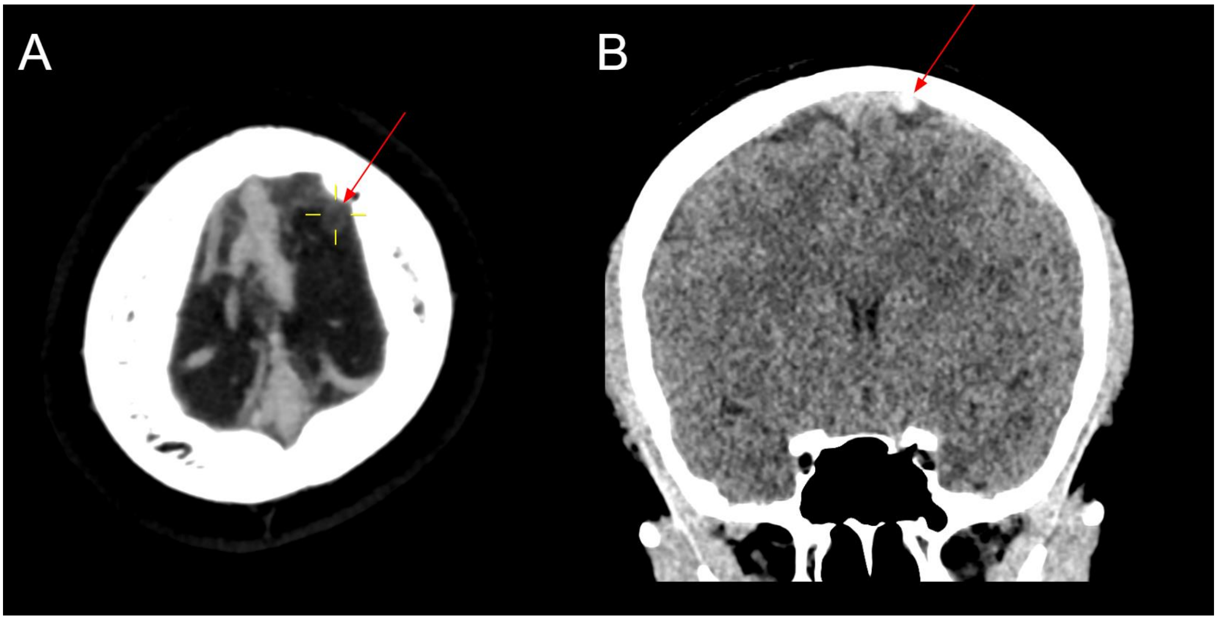

A. CT venography with red arrow indicating lack of filling at the site of the thrombus
B. Non-enhanced CT with red arrow indicating high attenuating thrombus

ID 11.

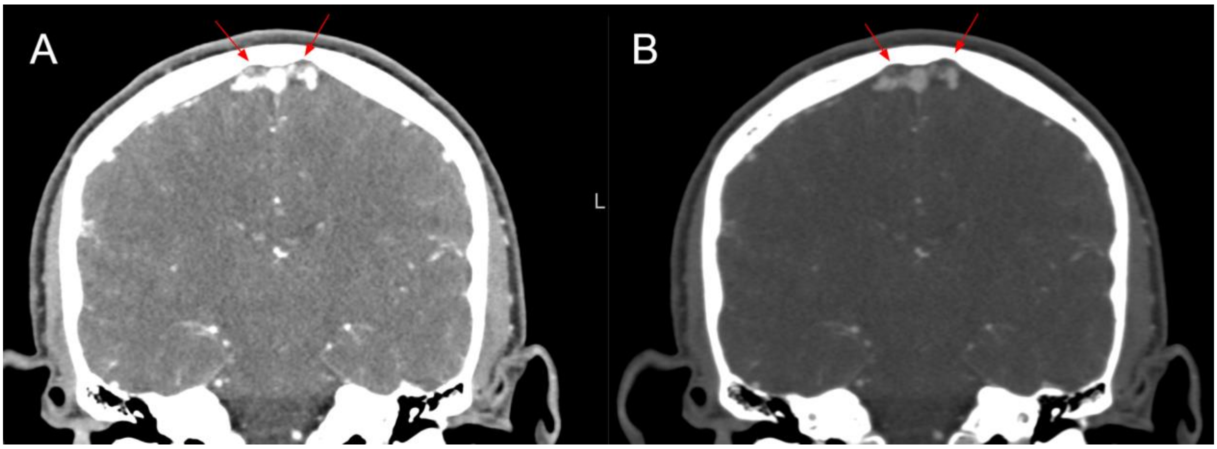

A. CT venography with red arrows indicating lack of filling at the site of the thrombus
B. CT venography with red arrows indicating lack of filling at the site of the thrombus

ID 12.

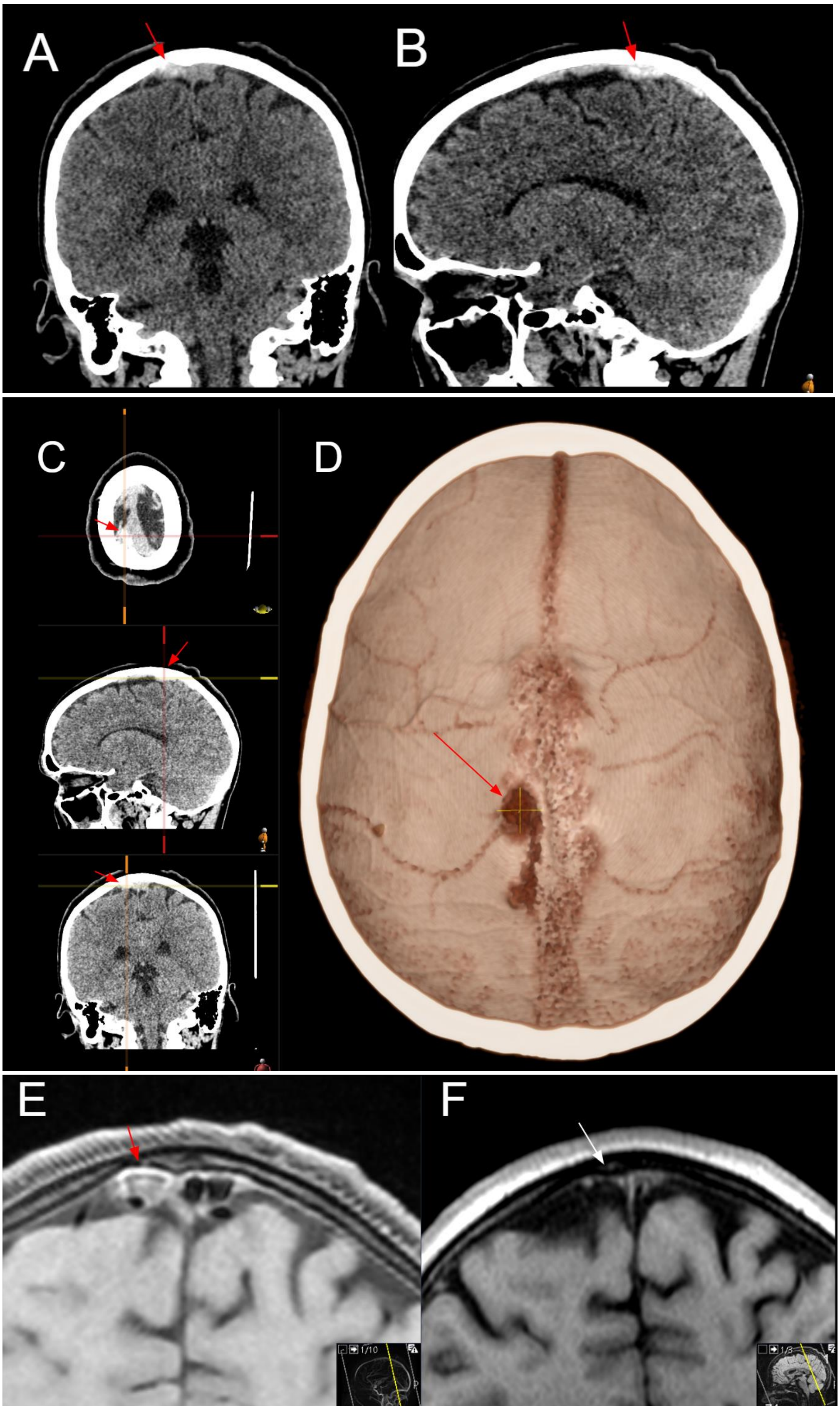

A. Non-enhanced CT with red arrow indicating high attenuating thrombus
B. Non-enhanced CT with red arrow indicating high attenuating thrombus

C. Non-enhanced CT in multiplanar reformation with red arrow indicating high attenuating thrombus
D. Volume rendering of non-enhanced CT with red arrow indicating high attenuation at the site of the thrombus
E. MRI T1 with red arrow indicating high signal in thrombus
F. MRI T1 at follow-up with white arrow indicating involution of thrombus
